# Unveiling trends in malignant neoplasms of bone and articular cartilage mortality in the United States (1999–2023): a CDC WONDER perspective

**DOI:** 10.3389/fonc.2026.1748685

**Published:** 2026-05-08

**Authors:** Bo Chen, Xiangyi Li

**Affiliations:** 1 Department of Orthopaedics, The Second People's Hospital of Changzhi City, Shanxi, China; 2 Graduate School, Changzhi Medical College, Shanxi, China

**Keywords:** articular cartilage, bone, CDC WONDER, epidemiology, malignant neoplasm, mortality

## Abstract

**Background:**

Malignant neoplasms of bone and articular cartilage (MNBAC) are rare but highly fatal cancers contributing to global cancer mortality. This study analyzed demographic and regional patterns of MNBAC mortality in the United States from 1999 to 2023 to identify high-risk populations for targeted interventions.

**Methods:**

We extracted mortality data from the CDC WONDER database using ICD-10 codes C40-C41. Crude and age-adjusted mortality rates (AAMRs) per 100,000 population were calculated by gender, census region, race, and urbanization using R software (version 4.4.2). Joinpoint regression was employed to evaluate annual percentage changes (APCs).

**Results:**

From 1999 to 2023, MNBAC mortality trends varied across demographic groups. The South showed the highest AAMR (0.60 in 1999, 95% CI: 0.54 to 0.66; 0.68 in 2023, 95% CI: 0.63 to 0.73), while the West demonstrated the steepest increase (AAPC = 1.33, 95% CI: 0.92 to 1.74). Age analysis revealed the most significant crude mortality rate increases among individuals aged ≥75 years (AAPC = 0.90 for 75-84; AAPC = 1.89 for 85+), with accelerated growth after 2012 (APC = 2.52 and 4.34, respectively). Among racial groups, Hispanics showed the fastest AAMR increase (AAPC = 1.01, 95% CI: 0.36 to 1.66), though non-Hispanic whites maintained the highest AAMR (0.68, 95% CI: 0.64 to 0.72). Metropolitan areas exhibited greater AAMR increases than non-metropolitan areas (AAPC = 0.84 vs. 0.27). Males consistently showed higher AAMRs than females (0.65 vs. 0.45 in 1999; 0.72 vs. 0.53 in 2023; **Figure 1**). Overall AAMR increased marginally from 2014 to 2018 (APC = 4.26, 95% CI: 0.00 to 8.69) before stabilizing.

**Conclusion:**

Significant disparities exist in MNBAC mortality, with elevated rates among males, Southerners, and non-Hispanic whites. Notable increases occurred in the West, among those aged ≥75 years, Hispanics, and metropolitan residents. These findings underscore the need for targeted interventions to improve outcomes and reduce mortality.

**Figure 1:**
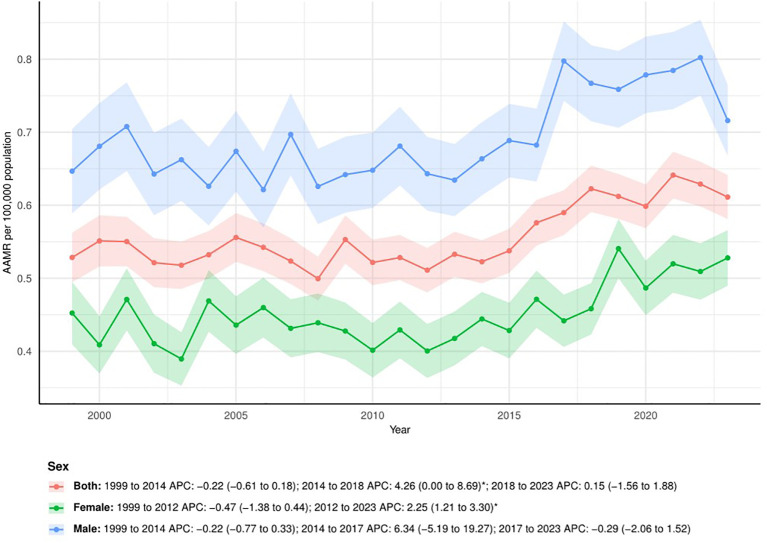
The trend of AAMRs stratified by gender. The * denotes the APC that was found to be statistically at α= 0.05.

## Introduction

Cancer is the second leading cause of death in developed countries and the third leading cause of death among adults in developing countries (1). Primary bone tumors are rare, but they are a major contributor to mortality and morbidity in cancer patients (1). Bone cancer (BC) is a highly heterogeneous group of rare cancers, comprising over 50 different histologies (2). As a debilitating and metastasizing malignancy involving the musculoskeletal system, BC causes significant disability and mortality and affects all age groups compared to common cancer types (3). According to the International Classification of Diseases 10th version (ICD-10), codes C40-C41 refer to primary malignant neoplasms originating from bone and articular cartilage, accounting for approximately 0.2% of all cancers and classified as one of the rare cancers (3). The most common subtypes of bone cancer are chondrosarcoma, osteosarcoma, chordoma, and Ewing sarcoma (3). Osteosarcoma is a primary malignant tumor of the skeleton characterized by the direct formation of bone or bone-like tissue by tumor cells (4, 5). It is the most common type of primary bone tumor, with an annual incidence of 1–3 cases per million individuals, accounting for ~0.2% of malignant tumors and ~15% of primary bone tumors (6–9). The most common sites of tumor development are the long bones, typically the distal femur or proximal humerus; occurrences at other sites are rare (10, 11). Hematogenous metastasis in osteosarcoma occurs early, with a high incidence, rapid progression, high mortality, and great difficulty in treatment (12, 13). Chondrosarcoma (CS) is a collective term for a group of heterogeneous, generally slow-growing primary malignant bone tumors, characterized by the formation of hyaline cartilaginous neoplastic tissue (14). Primarily affecting adults, it is the second most common primary solid bone tumor after osteosarcoma (15), with an annual incidence of approximately 3 new cases per 10^6^ population (16). Although the etiology and biological mechanisms of most bone cancers remain unclear, studies have suggested that tumor growth and development, germline genetic factors, somatic alterations, environmental exposure, and socioeconomic status are all associated with the predisposition to and development of BC (3). With the rapid transitions in lifestyle behaviors, socioeconomic status, and healthcare systems in modern society, changes in the spectrum of cancer mortality for several common cancer types have been widely reported (3). However, it remains poorly understood whether rare cancers such as malignant tumors of bone and articular cartilage follow similar or different epidemiological patterns. Therefore, large-scale, population-based studies on the epidemiological changes of bone cancer are still needed to better understand the disease and provide a basis for formulating targeted prevention and treatment strategies.

## Methods

### Population and study selection

We accessed the CDC WONDER database (https://wonder.cdc.gov/), which compiles death certificate data from 1999 to 2023, to calculate mortality rates for patients with malignant bone and articular cartilage tumors. Diagnoses were identified using ICD-10 codes C40-C41 (C40: malignant neoplasm of bone and articular cartilage of limbs; C41: malignant neoplasm of bone and articular cartilage of other and unspecified sites), classified as the underlying cause of death. This study aimed to examine mortality trends of malignant bone and articular cartilage tumors in the adult population; therefore, the study population comprised individuals aged 25 to 85 years and older. Since this observational study used publicly available de-identified datasets, Institutional Review Board approval was deemed unnecessary. This study followed the 22-item Strengthening the Reporting of Observational Studies in Epidemiology (STROBE) guidelines to ensure credibility and reliability.

### Data collection

We gathered data on demographics, geographic regions, population sizes, and years. Demographic details were categorized by age (25-34, 35-44, 45-54, 55-64, 65-74, 75-84, 85+ years), sex (male, female), census region (Northeast, Midwest, South, West), urbanization (metropolitan, non-metropolitan) and race (Hispanic, Non-Hispanic Black, Non-Hispanic White). Non-Hispanic Other was excluded due to incomplete data. The analysis was based on death certificate information, a method validated by previous studies. The 2013 National Center for Health Statistics Urban-Rural Classification Scheme was used to stratify counties into metropolitan and nonmetropolitan categories. Furthermore, according to US Census Bureau classification, the US was partitioned into four separate regions. Data cells with <10 deaths were suppressed by CDC WONDER and were not included in trend analyses. Bridged-race population estimates were used as denominators for rate calculations.

### Statistical analysis

We conducted a detailed statistical analysis of mortality rates for malignant neoplasms of bone and articular cartilage from 1999 to 2023, including both crude and age-adjusted rates. Ninety-five percent confidence intervals (CIs) were presented for the following mortality rate categories: sex, census region, race, urbanization level, and age group. Crude mortality rates were calculated by dividing the total number of deaths each year by the corresponding US population. Age-adjusted mortality rates (AAMRs) were standardized to the 2000 US Standard Population using the direct method to account for differences in age distribution over time (17, 18).

To analyze nationwide changes in mortality rates, the annual percent change (APC), average annual percent change (AAPC), and their 95% CIs for AAMRs or crude mortality rates were calculated using the Joinpoint Regression Program (Version 5.4.0.0). Joinpoint regression is particularly suitable for this type of analysis because it can detect multiple changes in trend direction, rather than assuming a constant rate of increase or decrease (18). This is crucial for accurately identifying periods of significant change, such as sudden increases or decreases in mortality rates that may correspond to factors like medical advancements or changes in disease prevalence (18, 19). This statistical method uses a log-linear regression model to identify points where significant changes in trends occur, allowing precise location of shifts in mortality rates over time (18).

Joinpoint model specifications were as follows: Grid Search method with a minimum of 2 observations between joinpoints and 2 observations before the first/after the last joinpoint; maximum number of joinpoints: 4; model selection: Permutation Test with 4,499 permutations; overall significance level: α = 0.05 (two-sided); confidence intervals calculated using the parametric method with 5,001 resamples; AAPC calculated for the entire study period (1999–2023). A two-tailed t-test was used to determine the significance of slopes, thereby enabling classification of changes in mortality rates as increasing or decreasing (18). A p-value of less than 0.05 was considered statistically significant.

## Results

### Patterns of AAMRs for malignant bone and joint cartilage tumors stratified by gender

This study analyzed the mortality data of malignant bone and joint cartilage tumors in the United States from 1999 to 2023, revealing the trends and characteristics of mortality changes among different genders. The findings indicate that during the period from 1999 to 2023, the number of deaths among both males and females in the United States increased significantly, with the highest number of deaths among males (17,090) and a lower number among females (13,692). In terms of annual data, the number of deaths for each gender was relatively low in 1999, but by 2023, the number of deaths for both genders had increased. The percentage change analysis showed that the number of deaths among females increased the fastest (75.51%), followed by males (72.76%) and the overall population (74.04%). (Attachment, **Table 1**)

**Table 1:** Trends in deaths and AAMRs or crude mortality rates for malignant bone and joint cartilage tumors in the United States, 1999–2023.

Characteristic	Deaths			AAMRs/crude mortality rates		
	1999	2023	Percent change(%)	1999 (95%CI)	2023 (95%CI)	AAPC (95% CI)
Overall	940	1636	74.04	0.53 (0.49 to 0.56)	0.61 (0.58 to 0.64)	0.59 (-0.17 to 1.36
Sex						
Female	437	767	75.51	0.45 (0.41 to 0.50)	0.53 (0.49 to 0.57)	0.76 (0.12 to 1.41*
Male	503	869	72.76	0.65 (0.59 to 0.70)	0.72 (0.67 to 0.76)	0.56 (-0.87 to 2.01
Census region						
Northeast	151	233	54.30	0.43 (0.36 to 0.49)	0.48 (0.42 to 0.55)	0.37 (-0.10 to 0.84
Midwest	232	330	42.24	0.56 (0.49 to 0.63)	0.60 (0.53 to 0.66)	0.51 (-0.05 to 1.07
South	376	714	89.89	0.60 (0.54 to 0.66)	0.68 (0.63 to 0.73)	0.79 (0.38 to 1.21*
West	181	359	98.34	0.50 (0.43 to 0.57)	0.60 (0.54 to 0.67)	1.33 (0.92 to 1.74*
Race						
Hispanic	63	158	150.79	0.52 (0.38 to 0.69)	0.51 (0.43 to 0.59)	1.01 (0.36 to 1.66*
NH Black	87	151	73.56	0.51 (0.40 to 0.63)	0.55 (0.46 to 0.64)	0.64 (0.17 to 1.12*
NH White	770	1247	61.95	0.54 (0.50 to 0.58)	0.68 (0.64 to 0.72)	0.84 (-0.37 to 2.07
Urbanization#						
Metropolitan	706	1334	88.95	0.47 (0.44 to 0.51)	0.57 (0.53 to 0.60)	0.84 (0.30 to 1.39*
Nonmetropolitan	234	302	29.06	0.73 (0.64 to 0.83)	0.76 (0.67 to 0.85)	0.27 (-0.13 to 0.67
Age##						
25–34 years	87	105	20.69	0.22 (0.22 to 0.22)	0.23 (0.23 to 0.23)	0.49 (0.01 to 0.98*
35–44 years	91	97	6.59	0.20 (0.20 to 0.20)	0.22 (0.22 to 0.22)	0.49 (0.08 to 0.90*
45–54 years	130	113	-13.08	0.36 (0.36 to 0.36)	0.28 (0.28 to 0.28)	-0.83 (-3.23 to 1.63
55–64 years	107	249	132.71	0.45 (0.45 to 0.45)	0.59 (0.59 to 0.59)	0.77 (-0.11 to 1.66
65–74 years	182	375	106.04	0.99 (0.99 to 0.99)	1.08 (1.08 to 1.08)	0.84 (0.41 to 1.27*
75–84 years	219	394	79.91	1.79 (1.79 to 1.79)	2.15 (2.15 to 2.15)	0.90 (0.32 to 1.49*
85+ years	124	303	144.35	2.99 (2.99 to 2.99)	4.89 (4.89 to 4.89)	1.89 (1.11 to 2.67*

AAPC values with statistical significance are marked with *. #AAMR data for 2023 urbanization level categories were based on the 2020 classifications. AAPCs were calculated for the period 1999-2023. ##Age-specific mortality rates are crude mortality rates (not age-adjusted); corresponding AAPCs were derived from these crude rates. AAMR, age-adjusted mortality rate; CI,confidence interval; AAPC, average annual percent change.

Regarding AAMRs, upward trends were observed for both genders. The AAMR for females increased from 0.45 per 100,000 in 1999 (95% CI: 0.41 to 0.50) to 0.53 per 100,000 in 2023 (95% CI: 0.49 to 0.57). For males, the AAMR rose from 0.65 per 100,000 in 1999 (95% CI: 0.59 to 0.70) to 0.72 per 100,000 in 2023 (95% CI: 0.67 to 0.76). The overall AAMR for the population increased from 0.53 per 100,000 in 1999 (95% CI: 0.49 to 0.56) to 0.61 per 100,000 in 2023 (95% CI: 0.58 to 0.64). (**Table 1**)

The AAPCs further illustrated the changes in AAMRs among different genders. The AAPC for females was 0.76 (95% CI: 0.12 to 1.41), which indicated a significant upward trend. For males, the AAPC was 0.56 (95% CI: -0.87 to 2.01), and for the overall population, the AAPC was 0.59 (95% CI: -0.17 to 1.36), with no significant upward trend. (**Table 1**)

The APCs showed the following trends: For the overall population, the AAMR decreased non-significantly from 1999 to 2014 (APC = -0.22, 95% CI: -0.61 to 0.18), increased with marginal significance from 2014 to 2018 (APC = 4.26, 95% CI: 0.00 to 8.69), and remained stable from 2018 to 2023 (APC = 0.15, 95% CI: -1.56 to 1.88). For females, the AAMR decreased non-significantly from 1999 to 2012 (APC = -0.47, 95% CI: -1.38 to 0.44) and increased significantly from 2012 to 2023 (APC = 2.25, 95% CI: 1.21 to 3.30). For males, the AAMR decreased non-significantly from 1999 to 2014 (APC = -0.22, 95% CI: -0.77 to 0.33), increased non-significantly from 2014 to 2017 (APC = 6.34, 95% CI: -5.19 to 19.27), and decreased non-significantly from 2017 to 2023 (APC = -0.29, 95% CI: -2.06 to 1.52). (**Figure 1**)

### Patterns of AAMRs for malignant bone and joint cartilage tumors stratified by census region

This study analyzed the mortality data for malignant bone and joint cartilage tumors in the United States from 1999 to 2023, revealing the trends and characteristics of mortality changes across different regions. The findings indicate that during the period from 1999 to 2023, the number of deaths increased significantly in all four regions of the United States: the Northeast, Midwest, West, and South. The South had the highest number of deaths (13,004), while the Northeast had the lowest (4,710). In terms of annual data, the number of deaths in each region was relatively low in 1999, but by 2023, the number of deaths had increased in all regions. The percentage change analysis showed that the West had the fastest increase in the number of deaths (98.34%), followed by the South (89.89%), the Northeast (54.30%), and the Midwest (42.24%). (Attachment, **Table 1**)

Regarding AAMRs, regions exhibited upward trends. In the Northeast, the AAMR increased from 0.43 per 100,000 in 1999 (95% CI: 0.36 to 0.49) to 0.48 per 100,000 in 2023 (95% CI: 0.42 to 0.55). In the Midwest, the AAMR rose from 0.56 per 100,000 in 1999 (95% CI: 0.49 to 0.63) to 0.60 per 100,000 in 2023 (95% CI: 0.53 to 0.66). In the West, the AAMR increased from 0.50 per 100,000 in 1999 (95% CI: 0.43 to 0.57) to 0.60 per 100,000 in 2023 (95% CI: 0.54 to 0.67). In the South, the AAMR rose from 0.60 per 100,000 in 1999 (95% CI: 0.54 to 0.66) to 0.68 per 100,000 in 2023 (95% CI: 0.63 to 0.73), with the West and South showing the largest increases in AAMR. (**Table 1**)

The AAPCs further revealed the changes in AAMRs across regions. The West had an AAPC of 1.33 (95% CI: 0.92 to 1.74), and the South had an AAPC of 0.79 (95% CI: 0.38 to 1.21), both indicating significant upward trends. The Northeast (AAPC = 0.37, 95% CI: -0.10 to 0.84) and the Midwest (AAPC = 0.51, 95% CI: -0.05 to 1.07) showed non-significant upward trends. (**Table 1**)

**Figure 2:**
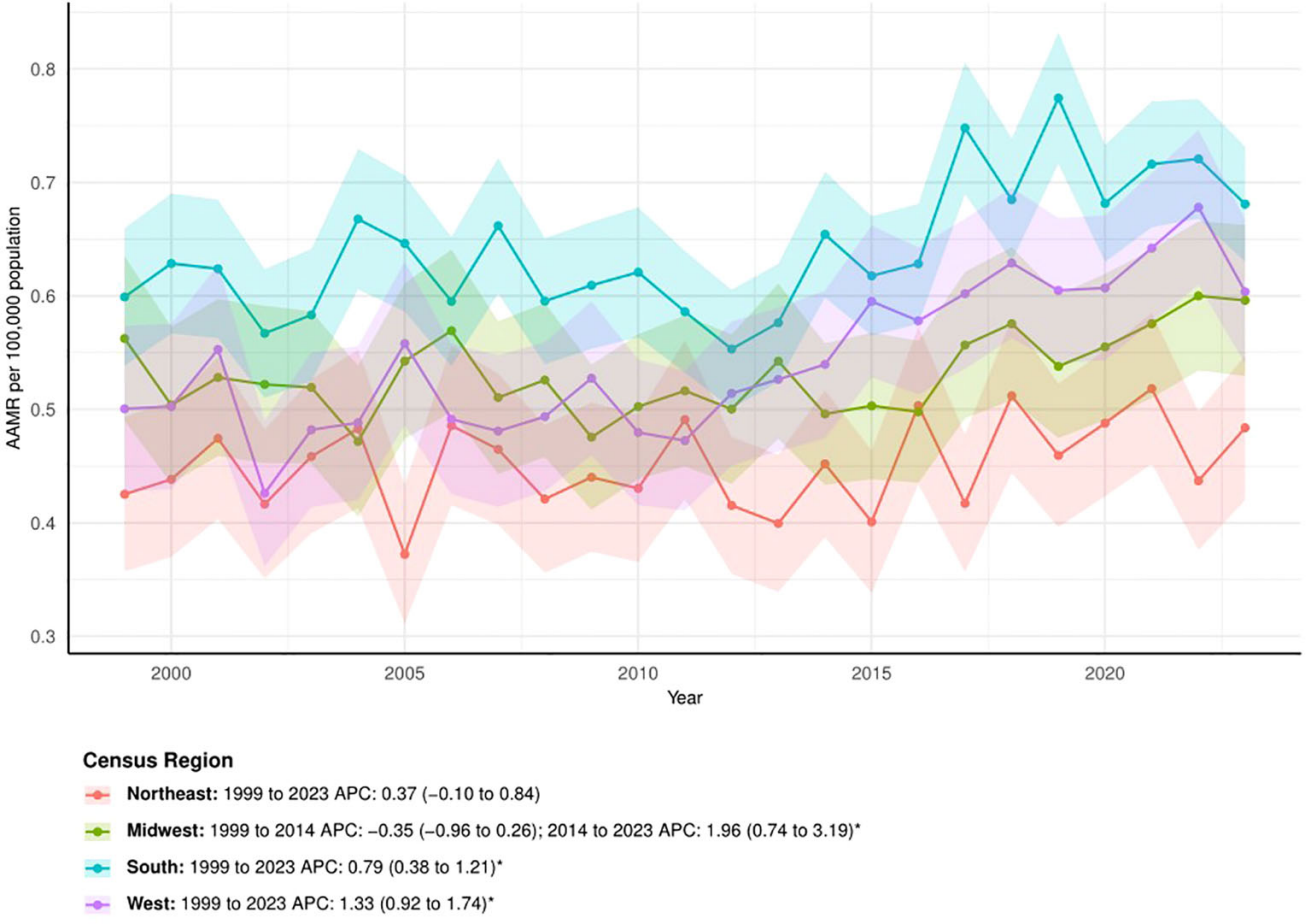
The trend of AAMRs stratified by census region. The * denotes the APC that was found to be statistically at α= 0.05.

The APCs revealed that the West had the most significant upward trend (APC = 1.33, 95% CI: 0.92 to 1.74), followed by the South (APC = 0.79, 95% CI: 0.38 to 1.21). The Northeast showed no statistically significant change (APC = 0.37, 95% CI: -0.10 to 0.84). In the Midwest, the AAMR increased significantly after 2014 (APC = 1.96, 95% CI: 0.74 to 3.19 for 2014–2023), which contrasted sharply with the relatively stable trend before 2014 (APC = -0.35, 95% CI: -0.96 to 0.26 for 1999–2014) (**Figure 2**).

### Patterns of AAMRs for malignant bone and joint cartilage tumors stratified by race

This study analyzed the mortality data for malignant bone and joint cartilage tumors among different races in the United States from 1999 to 2023, revealing the trends and characteristics of mortality changes across racial groups. The findings indicate that during the period from 1999 to 2023, the number of deaths increased significantly among non-Hispanic whites, non-Hispanic blacks, and Hispanics. The highest number of deaths was observed among non-Hispanic whites (23,849), while the lowest was among Hispanics (2,557). In terms of annual data, the number of deaths across all racial groups was relatively low in 1999, but by 2023, the number of deaths had increased in all groups. The percentage change analysis showed that the number of deaths among Hispanics increased the fastest (150.79%), followed by non-Hispanic blacks (73.56%) and non-Hispanic whites (61.95%). (Attachment, **Table 1**)

Regarding AAMRs, the AAMR for Hispanics decreased slightly from 0.52 per 100,000 in 1999 (95% CI: 0.38 to 0.69) to 0.51 per 100,000 in 2023 (95% CI: 0.43 to 0.59). For non-Hispanic blacks, the AAMR increased from 0.51 per 100,000 in 1999 (95% CI: 0.40 to 0.63) to 0.55 per 100,000 in 2023 (95% CI: 0.46 to 0.64). For non-Hispanic whites, the AAMR rose from 0.54 per 100,000 in 1999 (95% CI: 0.50 to 0.58) to 0.68 per 100,000 in 2023 (95% CI: 0.64 to 0.72). The AAMR for Hispanics remained relatively stable, while the AAMRs for non-Hispanic whites and non-Hispanic blacks showed larger increases. (**Table 1**)

The AAPCs further illustrated the changes in AAMRs across racial groups. Hispanics had an AAPC of 1.01 (95% CI: 0.36 to 1.66), and non-Hispanic blacks had an AAPC of 0.64 (95% CI: 0.17 to 1.12), both indicating significant upward trends. Non-Hispanic whites had a non-significant upward trend (AAPC = 0.84, 95% CI: -0.37 to 2.07). (**Table 1**)

**Figure 3:**
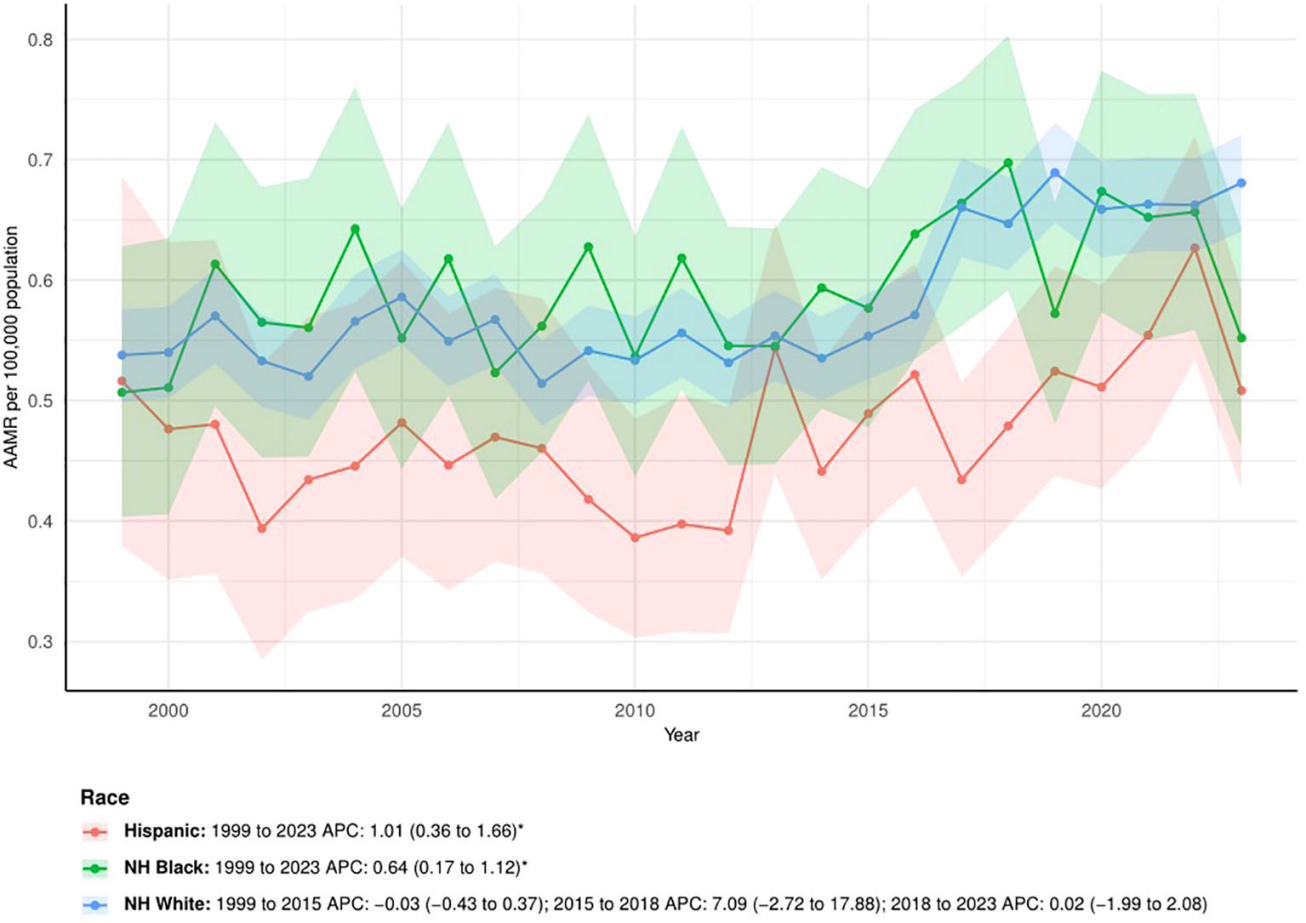
The trend of AAMRs stratified by race. The * denotes the APC that was found to be statistically at α= 0.05.

The APCs showed that AAMRs continued to rise among Hispanics (APC: 1.01, 95% CI: 0.36 to 1.66) and non-Hispanic blacks (APC: 0.64, 95% CI: 0.17 to 1.12). In contrast, the AAMR among non-Hispanic whites remained stable from 1999 to 2015 (APC: -0.03, 95% CI: -0.43 to 0.37), showed a non-significant upward trend from 2015 to 2018 (APC: 7.09, 95% CI: -2.72 to 17.88), and then stabilized again from 2018 to 2023 (APC: 0.02, 95% CI: -1.99 to 2.08). (**Figure 3**)

### Patterns of AAMRs for malignant bone and joint cartilage tumors stratified by urbanization

This study analyzed the mortality data for malignant bone and joint cartilage tumors in metropolitan and nonmetropolitan areas of the United States from 1999 to 2023, revealing the trends and characteristics of changes in the number of deaths. The findings indicate that during the period from 1999 to 2023, the number of deaths increased significantly in both nonmetropolitan and metropolitan areas. In terms of annual data, the number of deaths in each area was relatively low in 1999, but by 2023, the number of deaths had increased in both areas. The percentage change analysis showed that the number of deaths in metropolitan areas increased more rapidly (88.95%), compared with nonmetropolitan areas (29.06%). (Attachment, **Table 1**)

Regarding AAMRs, both metropolitan and nonmetropolitan areas exhibited upward trends. The AAMR in nonmetropolitan areas increased from 0.73 per 100,000 in 1999 (95% CI: 0.64 to 0.83) to 0.76 per 100,000 in 2023 (95% CI: 0.67 to 0.85), and in metropolitan areas, it rose from 0.47 per 100,000 in 1999 (95% CI: 0.44 to 0.51) to 0.57 per 100,000 in 2023 (95% CI: 0.53 to 0.60). (**Table 1**)

The AAPCs further illustrated the changes in AAMRs across the areas. The AAPC for metropolitan areas was 0.84 (95% CI: 0.30 to 1.39), indicating a significant upward trend, while the AAPC for nonmetropolitan areas was 0.27 (95% CI: -0.13 to 0.67), with no significant upward trend. (**Table 1**)

**Figure 4:**
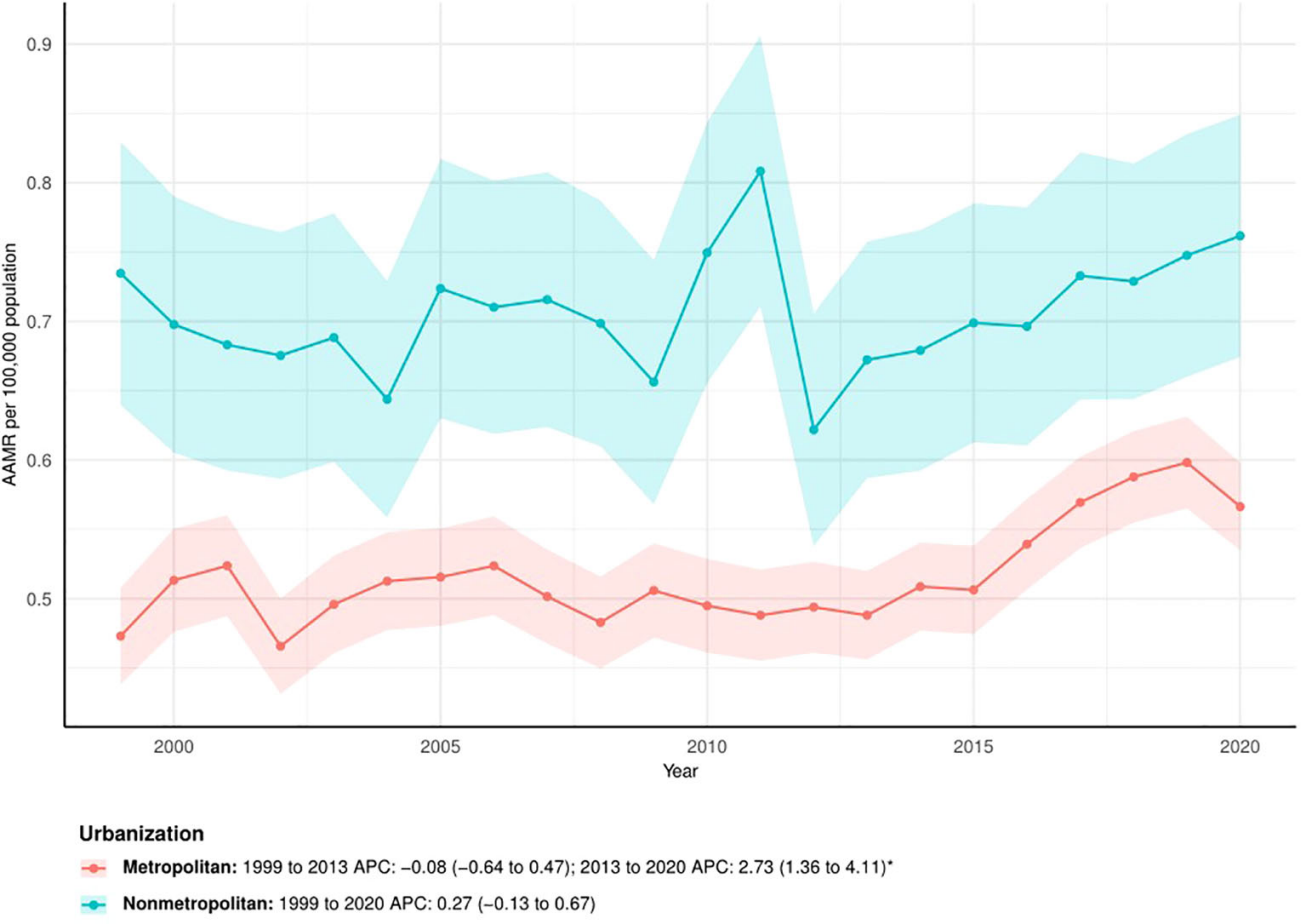
The trend of AAMRs stratified by urbanization. The * denotes the APC that was found to be statistically at α= 0.05.

The APCs showed that the AAMR in metropolitan areas remained stable from 1999 to 2013 (APC = -0.08; 95% CI: -0.64 to 0.47) but increased significantly from 2013 to 2020 (APC = 2.73; 95% CI: 1.36 to 4.11). In contrast, the AAMR in nonmetropolitan areas showed no significant change overall from 1999 to 2020 (APC = 0.27; 95% CI: -0.13 to 0.67) (**Figure 4**).

### Patterns of crude mortality rates for malignant bone and joint cartilage tumors stratified by age

This study conducted an in-depth analysis of the mortality data for malignant bone and joint cartilage tumors across different age groups in the United States from 1999 to 2023, revealing the trends and characteristics of changes in the number of deaths in each age group. The results showed that during the period from 1999 to 2023, the number of deaths increased significantly in all age groups. The highest number of deaths was observed in the 75-84 age group (6,708 deaths), while the lowest was in the 35-44 age group (2,204 deaths). In terms of annual data, the number of deaths in each age group was relatively low in 1999, but by 2023, the number of deaths had increased significantly in all age groups. The percentage change analysis showed that the number of deaths increased the fastest in the 85+ age group (144.35%), followed by the 55-64 age group (132.71%), the 65-74 age group (106.04%), and the 75-84 age group (79.91%). (Attachment, **Table 1**)

Regarding crude mortality rates, the crude mortality rate for the 65-74 age group increased from 0.99 per 100,000 in 1999 (95% CI: 0.99 to 0.99) to 1.08 per 100,000 in 2023 (95% CI: 1.08 to 1.08). For the 75-84 age group, the crude mortality rate rose from 1.79 per 100,000 in 1999 (95% CI: 1.79 to 1.79) to 2.15 per 100,000 in 2023 (95% CI: 2.15 to 2.15). For the 85+ age group, the crude mortality rate increased from 2.99 per 100,000 in 1999 (95% CI: 2.99 to 2.99) to 4.89 per 100,000 in 2023 (95% CI: 4.89 to 4.89). (**Table 1**)

The AAPCs further revealed the changes in crude mortality rates across age groups. The AAPC for the 85+ age group was 1.89 (95% CI: 1.11 to 2.67), indicating a significant upward trend. The AAPC for the 75-84 age group was 0.90 (95% CI: 0.32 to 1.49), and for the 65-74 age group, it was 0.84 (95% CI: 0.41 to 1.27), both showing significant upward trends. (**Table 1**)

**Figure 5:**
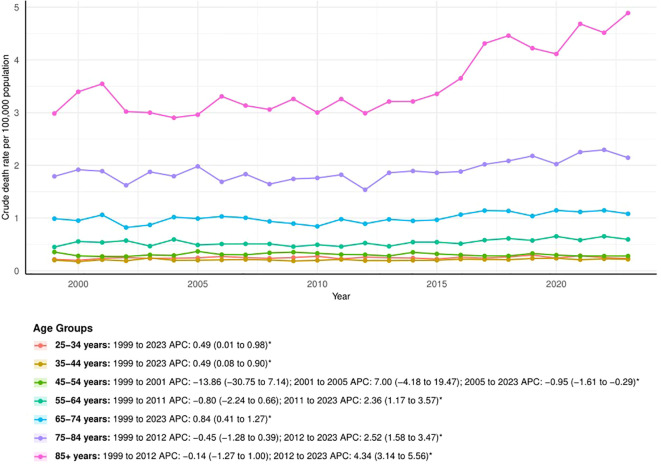
The trend of crude mortality rates stratified by age. The * denotes the APC that was found to be statistically at α= 0.05.

The APCs showed that crude mortality rates in younger groups (25-44 years) exhibited marginally significant upward trends (APC = 0.49, 95% CI: 0.01 to 0.98; APC = 0.49, 95% CI: 0.08 to 0.90). For middle-aged groups (45-54 years), the crude mortality rate decreased after 2005 (APC = -0.95, 95% CI: -1.61 to -0.29). In older groups (≥55 years), the crude mortality rate generally increased, with more pronounced trends in older ages. Notably, individuals aged 75 and above showed an accelerated upward trend after 2012 (75-84 years: APC = 2.52, 95% CI: 1.58 to 3.47; 85+ years: APC = 4.34, 95% CI: 3.14 to 5.56). (**Figure 5**)

## Discussion

This study is based on the mortality data of malignant bone and joint cartilage tumors in the United States from 1999 to 2023. It analyzes the mortality trends of different regions, races, age groups, urban and non-urban areas, and genders. It reveals significant dynamic changes in these trends, providing important evidence for future research and prevention strategies. The mortality from malignant bone and joint cartilage tumors increased in all regions of the United States. The southern region had the highest mortality, while the northeastern region had the lowest. The western region had the fastest growth rate. These regional disparities are consistent with documented imbalances in economic and medical resources across the United States. The geographical differences that have been exacerbated over time often reflect the distribution of poverty across the country (20). The inequality in health-care access has been increasingly widened due to the differences among states in the expansion of Medicaid and other initiatives to increase health-care coverage (20–22). The mortality increased in races, with the fastest growth in Hispanics, while non-Hispanic whites had the highest AAMR. Jawad et al. (23) also showed racial differences in their study. Their research indicated that whites had the highest incidence rate, followed by Asians/Pacific Islanders, while African-Americans had the lowest incidence rate. The incidence rate difference between whites and African-Americans was as high as 9-fold. Only the incidence rate of Ewing’s sarcoma in whites showed an upward trend. These racial disparities parallel patterns of wealth inequality, which leads to exposure to risk factors and barriers to high-quality prevention, early detection, and treatment (20).

The mortality increased in age groups. The 75-84-year-old group had the highest crude mortality rate while the 35-44-year-old group had the lowest. The 85-plus-year-old group had the fastest growth rate, consistent with established age-related increases in mortality risk. This is similar to the findings of some other studies. Fukushima et al. (24) showed that adolescence and young age were not predictors of poor cancer survival, while older age (≥65 years) was a predictor of poor cancer survival in patients with osteosarcoma and chondrosarcoma. Savage et al. (25) showed that survival rates dropped sharply after the age of 50. The survival rate of patients decreased from about 50% in their 50s to 17% in their 60s and only 11% in their 80s. Data from the SEER program in the United States from 1973 to 2004 showed that the 5-year relative survival rate was 61.6% for young patients with osteosarcoma, 58.7% for middle-aged patients, and only 24.2% for patients over 60 years old (25, 26). Elderly patients may have unique tumor biology (25). Paget’s disease is a relatively common metabolic bone disease, which is more common in the elderly (25, 27, 28). It is characterized by extreme enhancement of the bone remodeling process caused by abnormal regulation of osteoclasts (25). It is estimated that about half of the reported osteosarcoma cases in the elderly population are associated with Paget’s disease (25). It has been reported that osteosarcoma in the elderly (average age 65 years) has unique genetic changes. In addition to TP53 or LSAMP mutations, it is also characterized by H3F3A mutations and has a distinct DNA methylation profile (29, 30). This unique tumor biology may make the elderly more resistant to chemotherapy. Tsuchie et al. (31) also confirmed this view. Their study showed that primary osteosarcoma in elderly patients was characterized by a high incidence of axial bone involvement, low use of chemotherapy, and chemoresistance. Additionally, elderly patients often have comorbidities, which can limit their medication and surgical options.

The mortality increased in both metropolitan and non-metropolitan areas, but the growth rate was faster in metropolitan areas. Environmental exposures such as radiation and pollution are more prevalent in urban areas and have been hypothesized as contributing factors (32, 33). On the other hand, the widespread use of advanced diagnostic technologies, such as CT and MRI imaging, has enhanced the detection capacity and contributed to more accurate diagnosis (33). These factors may have contributed to improved case ascertainment and data completeness, partially accounting for the observed increase. The mortality increased in both males and females, but the AAMR of males was always higher than that of females. This is similar to the findings of some other studies. Petrilli et al. (34, 35) analyzed 92 patients with non - metastatic osteosarcoma and found that male gender was a predictor of tumor recurrence and a poor prognostic indicator for death. The reason for the increased susceptibility in males is still unclear, but it may reflect the complex interactions among environmental exposure, endogenous hormones, and other influencing factors to some extent (20). Bielack et al. (34, 36) attributed the differences between males and females to the poorer response of male osteosarcoma patients to neoadjuvant chemotherapy based on 1,702 osteosarcoma patients. Wagle et al. (34, 37) reported that knocking down androgen receptor coactivators and androgen receptors could inhibit the proliferation-related signaling in osteosarcoma cells and reduce cell proliferation. Fang et al. (38) observed that 17β-estradiol (E2) significantly affected the proliferation of osteosarcoma cells. High-dose E2 treatment could inhibit the proliferation, migration, and invasion of osteosarcoma cells. These biological mechanisms warrant further investigation as potential contributors to sex disparities in AAMR.

In summary, this study revealed an upward trend in mortality from malignant bone and articular cartilage tumors in the United States from 1999 to 2023. The observed trends are consistent with demographic shifts, regional disparities, and environmental factors, though causality cannot be established from mortality data alone. Future research incorporating incidence data is warranted to elucidate the drivers of these trends.

### Limitations

Our study has several limitations that should be acknowledged. Data limitations: The study is based solely on mortality data from death certificates, without information on tumor histology, treatment progress, socioeconomic factors, or lifestyle. Malignant neoplasms of bone and articular cartilage (ICD-10: C40-C41) comprise heterogeneous subtypes including osteosarcoma, chondrosarcoma, and Ewing sarcoma, which differ in age distribution, prognosis, and molecular characteristics. Our inability to stratify by histology may mask subtype-specific trends. Diagnostic limitations: Primary bone tumors may be misdiagnosed as metastatic carcinomas or benign lesions, particularly in elderly patients, and vice versa, potentially biasing mortality trends. Incidence versus survival: Using mortality data alone, we cannot distinguish whether observed increases reflect rising incidence, declining survival, or improved case detection. These mechanisms have different public health implications, but our data do not permit their differentiation. Lack of causal relationships: While we observed demographic and geographic disparities, we cannot attribute these to specific risk factors or healthcare variations. Our explanations remain speculative and warrant validation in studies with individual-level data. Geographic limitations: The study is limited to data from the United States and may not fully reflect the situation in other countries or regions, as different countries have different medical systems, socioeconomic conditions, and population structures. Future research should further explore the underlying factors contributing to changes in mortality rates, use multiple-source data to improve data integrity, and thereby gain a more comprehensive understanding of the trends in mortality rates for malignant bone and joint cartilage tumors.

## Conclusions

This study analyzed mortality data for malignant bone and joint cartilage tumors in the United States from 1999 to 2023. It found that mortality counts and rates increased across different regions, races, age groups, metropolitan and nonmetropolitan areas, and genders. The most pronounced increases were observed in the western region, the elderly population, Hispanics, and metropolitan areas. The AAMR of males was higher than that of females. Future research should further explore the factors involved and develop targeted interventions for high-risk populations. For example, it is necessary to enhance health monitoring and early screening for the elderly, optimize the allocation of medical resources in metropolitan areas, and conduct targeted health-education activities in the western region to cope with the burden of this disease.

## Data Availability

Publicly available datasets were analyzed in this study. This data can be found here: Repository: CDC WONDER Accession/URL: https://wonder.cdc.gov.
